# Characterization of *Plasmodium falciparum* genes associated with drug resistance in Hodh Elgharbi, a malaria hotspot near Malian–Mauritanian border

**DOI:** 10.1186/s12936-017-1791-2

**Published:** 2017-04-05

**Authors:** Mohamed Salem Ould Ahmedou Salem, Khadijetou Mint Lekweiry, Houssem Bouchiba, Aurelie Pascual, Bruno Pradines, Ali Ould Mohamed Salem Boukhary, Sébastien Briolant, Leonardo K. Basco, Hervé Bogreau

**Affiliations:** 1 Unité de Recherche «Génomes et Milieux», Faculté des Sciences et Techniques, Université de Nouakchott Al-Aasriya, Nouveau campus universitaire, BP 5026, Nouakchott, Mauritania; 2 Unité Parasitologie et entomologie, Département des maladies infectieuses, Institut de Recherche Biomédicale des Armées, Marseille, France; 3 Unité de Recherche sur les Maladies Infectieuses et Tropicales Emergentes (URMITE), Aix-Marseille Université, UM 63, CNRS 7278, IRD 198, Inserm 1095, AP-HM, Institut Hospitalo-Universitaire (IHU) - Méditerranée Infection, 19-21 Boulevard Jean Moulin, 13005 Marseille, France; 4 Centre national de référence pour le paludisme, Marseille, France

**Keywords:** *Plasmodium falciparum*, Drug resistance, Chloroquine, Amodiaquine, Antifolate drugs, Lumefantrine, Artemisinin-based combination therapy, Mauritania, Cross-border malaria

## Abstract

**Background:**

A malaria hotspot in the southeastern region of Mauritania, near the Malian border, may hamper malaria control strategies. The objectives were to estimate the prevalence of genetic polymorphisms associated with drug resistance in *Plasmodium falciparum* isolates and establish baseline data.

**Methods:**

The study was conducted in two malaria-endemic areas in Hodh Elgharbi, situated in the Malian–Mauritanian border area. Blood samples were collected from symptomatic patients. Single nucleotide polymorphisms in *Pfcrt*, *Pfmdr1*, *Pfdhfr*, and *Pfdhps* were genotyped using PCR-restriction fragment length polymorphism, DNA sequencing and primer extension. The *Pfmdr1* gene copy number was determined by real-time PCR.

**Results:**

Of 280 *P. falciparum*-infected patients, 193 (68.9%) carried the *Pfcrt* 76T mutant allele. The *Pfmdr1* 86Y and 184F mutations were found in 61 (23.1%) of 264 isolates and 167 (67.6%) of 247 samples that were successfully genotyped, respectively. *Pfmdr1* mutant alleles 1034C, 1042D and 1246Y were rarely observed. Of 102 *P. falciparum* isolates analysed, ten (9.8%) had more than one copy of *Pfmdr1* gene. The prevalence of isolates harbouring at least triple mutant *Pfdhfr* 51I, 59R, 108 N/T was 42% (112/268), of which 42 (37.5%) had an additional *Pfdhps* 437G mutation. The *Pfdhps* 540E mutation was observed in four isolates (1.5%), including three associated with *Pfdhfr* triple mutant. Only two quintuple mutants (*Pfdhfr*-51I-59R-108N *Pfdhps*-437G-540E) were observed.

**Conclusions:**

The observed mutations in *Pfdhfr*, *Pfdhps*, *Pfmdr1*, and *Pfcrt* may jeopardize the future of seasonal malaria chemoprevention based on amodiaquine-sulfadoxine-pyrimethamine, intermittent preventive treatment for pregnant women using sulfadoxine-pyrimethamine, and treatment with artesunate-amodiaquine. Complementary studies should be carried out to document the distribution, origin and circulation of *P. falciparum* populations in this region and more widely in the country to assess the risk of the spread of resistance.

## Background

Before 2006, chloroquine and sulfadoxine-pyrimethamine were the first- and second-line drugs in Mauritania, respectively. The current anti-malarial treatment policy in Mauritania is based on artesunate-amodiaquine and artemether-lumefantrine as the first- and second-line treatment of uncomplicated malaria, respectively, regardless of *Plasmodium* spp. [1]. The efficacy and tolerance of artesunate-amodiaquine treatment have been confirmed in a recent clinical study conducted in southern Mauritania [2]. The Mauritanian Ministry of Health also recommends the use of sulfadoxine-pyrimethamine (SP) for intermittent preventive treatment to prevent malaria during pregnancy, particularly during the first and second pregnancies [3]. Due to the scarcity of clinical data on anti-malarial drug efficacy in Mauritania, molecular markers of drug resistance are convenient surrogate indicators to monitor and detect emergence of drug-resistant *Plasmodium falciparum*.

Dihydrofolate reductase (DHFR) and dihydropteroate synthase (DHPS) are known targets of pyrimethamine and sulfadoxine, respectively [4]. These drugs specifically inhibit the enzymes of the folate pathway to kill the parasites. *P. falciparum* chloroquine resistance transporter (*Pfcrt*) and *P. falciparum* multidrug resistance gene 1 (*Pfmdr1*) encode membrane transporters that have been associated with resistance to chloroquine and amodiaquine (*Pfcrt*) or chloroquine, amodiaquine, and amino-alcohols (mefloquine, lumefantrine; *Pfmdr1*). The mode of action of chloroquine and other quinoline-like drugs involves an interference with the plasmodial haem metabolism in the digestive vacuole. Chloroquine-resistant *P. falciparum* parasites either diminish the influx of chloroquine into the food vacuole or enhance efflux of chloroquine from the food vacuole, or both, resulting in a decreased drug accumulation within the food vacuole [5]. Distinct point mutations in *Pfdhfr*, *Pfdhps* and *Pfcrt* confer resistance to pyrimethamine, sulfadoxine and chloroquine/amodiaquine, respectively. Some studies have suggested that point mutations in *Pfmdr1* are associated with resistance to quinoline-like drugs and artemisinin derivatives, but their role is not yet well established [6, 7]. Moreover, it has been reported that treatment with artemether-lumefantrine selects for the *pfmdr1* wild-type N86 allele [8–10]. *Pfmdr1* amplification resulting in multiple gene copies is associated with resistance to amino alcohols (mefloquine and lumefantrine) [4, 11]. Recent studies have demonstrated that mutations in Kelch propeller 13 is directly associated with artemisinin resistance [12].

Hodh Elgharbi region is one of the eight Mauritanian administrative regions where malaria transmission is seasonal, with the peak occurring in September and October. Several studies have shown that *P. falciparum* is the predominant malaria species in this region and that 30–83% of febrile patients are infected with malaria parasites during the transmission season [2, 13–16]. In Hodh Elgharbi region, the northern Saharo–Sahelian area that borders the Saharan desert has been considered as malaria-free while the southern Sahelian part of the region is classified as holo-endemic [13]. The Malian–Mauritanian border is particularly vulnerable to the spread of malaria parasites with the influx of war refugees from the Malian side of the frontier. The problem of human population movement is further compounded by the presence of populations leading a nomadic lifestyle in this agropastoral area. The situation in Mali near the border area is quite similar in terms of malaria epidemiology and chloroquine resistance [17, 18].

The objective of the present study was to compare the prevalence of *Pfcrt*, *Pfmdr1, Pfdhfr*, and *Pfdhps* mutations and the copy number of *Pfmdr1* gene in *P. falciparum* isolates collected in two areas with different malaria endemicity in Hodh Elgharbi region after the introduction of artemisinin-based combination therapy (ACT) in 2006, in order to establish a baseline database for monitoring drug-resistant *P. falciparum* in the malaria hotspot in the Malian–Mauritanian border area.

## Methods

### Study area

The study was conducted in Hodh Elgharbi, which covers a surface area of 53,400 sq km and is composed of four districts (Aioun (the provincial capital), Kobeni, Tamchekett, Tintane). Kobeni city is located about 18 km from the Malian–Mauritanian border. Aioun and Tintane are located approximately 103 and 174 km to the north and northwest of Kobeni city, respectively. The population of Hodh Elgharbi is approximately 294,109 inhabitants [19]. The location and Saharo–Sahelian (Aioun and Tintane) and Sahelian (Kobeni) climates were described in an earlier study [16]. Human activities are dominated by livestock rearing (mainly cattle, sheep, and goats) and agriculture (mainly millet). There are five public health centres in Hodh Elgharbi, one health centre in each of its four districts and one hospital in Aioun.

### Patients and blood sample collection

Between September and October 2010, corresponding to the peak season of malaria transmission, all febrile patients were enrolled after informed written consent. A rapid diagnostic test for malaria (SD Bioline *P. falciparum* histidine-rich protein II and *Plasmodium vivax* plasmodial lactate dehydrogenase antigen rapid diagnostic test; Standard Diagnostics, Inc, Yongin, Republic of Korea) was performed, and Giemsa-stained thin and thick blood films were prepared and examined under the microscope. Fifty µl of blood sample were spotted directly on Whatman 3MM filter paper, dried, and stored at room temperature for molecular analysis.

### Genetic characterization of *Plasmodium falciparum* isolates

Total genomic DNA of each isolate, including human DNA, was extracted using the MaxMag™DNA Multi-Sample kit (Applied Biosystems, Warrington, UK) following the manufacturer’s instructions. All molecular experiments were performed at the parasitology laboratory in Marseille, France in 2012–2013.

### *Pfcrt* single-nucleotide polymorphisms

Codon 76 (K76T, PlasmoDB single nucleotide polymorphism (SNP) ID: Pf3D7_07_v3: 403,625) was genotyped by restriction fragment length polymorphism (RFLP) as detailed elsewhere [20]. *Pfcrt* (PF3D7_0709000) was amplified by semi-nested PCR with fluorescent end-labelled primers (Table 1). First and second rounds of PCR had the same cycling conditions: 94 °C for 2 min, 10 cycles of 94 °C × 20 s, 50 °C × 20 s and 60 °C × 30 s, 35 cycles of 94 °C × 20 s, 45 °C × 20 s and 60 °C × 30 s, and a final 5-min extension step at 60 °C. The 6-FAM-labelled product (1 µl) was digested with 1.2 units of *Apo*I (New England Biolabs, Evry, France) at 50 °C for 3 h in a 30 µl reaction. Labelled and restricted products were diluted 100× and detected on ABI 3130XL capillary sequencer (Applied Biosystems) using Genescan 120 LIZ^®^ size standards. Genomic DNA from *P. falciparum* reference clones 3D7/unknown origin (wild-type) and W2/Indochina (mutant) was used as positive controls, and water and human DNA were used as negative controls.

**Table 1:** *Pfdhfr*, *Pfdhps*, *Pfmdr1* and *Pfcrt* PCR primer sequences used in amplification reactions

Gene	Protocol step	Primers	Primers sequences
*Pfdhfr*	PCR I	F	5′-TTC TCC TTT TTA TGA TGG AAC AAG T-3′
		R	5′ ATA TTT GAA AAT CAT TTG GAT GTA TAG-3′
	PCR II	F	5′-GTT GAA CCT AAA CGT GCT GT-3′
		R	5′-TTC ATC ATG TAA TTT TTG TTG TG-3′
	Multiplex	*Pfdhfr51F*	5′-AGG AGT ATT ACC ATG GAA ATG TA-3′
		*Pfdhfr16R*	5′-GAC TGA CTC TCA TTT TTG CTT TCA ACC TTA CAA CAT-3′
		*Pfdhfr108F*	5′-GAC TGA CTA CAA AAT GTT GTA GTT ATG GGA AGA ACA A-3′
		*Pfdhfr164R*	5′-CTG ACT GAC TGA CTG ACT AAT TCT TGA TAA ACA ACG GAA CCT CCT A-3′
		*Pfdhfr59R*	5′-CTG ACT GAC TGA CTG ACT GAC TTG ATT CAT TCA CAT ATG TTG TAA CTG CAC-3′
*Pfdhps*	PCR I	F	5′-TGC TTA AAT GAT ATG ATA CCC GAA TAT AAG-3′
		R	5′ TCC ACC TGA AAA GAA ATA CAT AAA T-3′
	PCR II	F	5′-GTT GAA CCT AAA CGT GCT GT-3′
		R	5′-TTC ATC ATG TAA TTT TTG TTG TG-3′
	Multiplex	*Pfdhps613R*	5′-TTG ATC ATT CAT GCA ATG GG-3′
		*Pfdhps540F*	5′-GAC TGA GGA AAT CCA CAT ACA ATG GAT-3′
		*Pfdhps581R*	5′-TAA GAG TTT AAT AGA TTG ATC ATG TTT CTT C-3′
		*Pfdh436*-*1F*	5′-GAC TGA CTA GTG TTA TAG ATA TAG GTG GAG AAT CC-3′
		*Pfdhps436*-*2R*	5′-GAC TGA CTG ACT GAC TTG GAT TAG GTA TAA CAA AAA GGA ICA-3′
		*Pfdhps437R*	5′-GAC TGA CTG ACT GAC TGA CTT TTT TGG ATT AGG TAT AAC AAA AGG A-3′
*Pfmdr1*	PfMDR1- 1	F	5′-AGA GAA AAA AGA TGG TAA CCT CAG-3′
		R	5′-ACC ACA AAC ATA AAT TAA CGG-3′
	PfMDR1- 2	F	5′-CAG GAA GCA TTT TAT AAT ATG CAT-3′
		R	5′-CGT TTA ACA TCT TCC AAT GTT GCA-3′
*Pfcrt*	PCR I	F	5′-GTT CTT GTC TTG GTA AAT GT-3′
		R	5′- CGG ATG TTA CAA AAC TAT AGT T-3′
	PCR II	F	5′-GTT CTT GTC TTG GTA AAT GT-3′
		R	**6-FAM**-5′- TAA ATG TGC TCA TGT GTT TA -3′

### *Pfmdr1* SNPs

*Pfmdr1* (PF3D7_0523000) genotyping was performed by Sanger’s method of DNA sequencing. Four pairs of primers were used to amplify two *Pfmdr1* fragments carrying five polymorphisms associated with drug resistant phenotype (Table 1). N86Y and Y184F were on the first fragment MDR1-1 (590 base pairs, bp) and S1034C, N1042D and D1246Y on fragment MDR1-2 (968 bp). The reaction mixture, PCR conditions, amplicons purification and sequencing were described in previous studies [20, 21].

### *Pfdhps and Pfdhfr* SNPs

*Pfdhps* and *Pfdhfr* genes were amplified by nested PCR and genotyped using a rapid primer extension method (Snapshot^®^), as previously described [22]. The following SNPs were determined: *Pfdhps* (PF3D7_0810800) S436F/A, A437G, K540E, A581G and A613S and *Pfdhfr* (PF3D7_0417200) A16V, N51I, C59R, S108 N/T, and I164L. Primer extension was performed and analysed by capillary electrophoresis on polyacrylamide gels using ABI 3130XL sequencer (Applied Biosystems). Electrophoregram was interpreted using Genemapper^®^ 4.0 software (Applied Biosystems, Carlsbad, CA, USA).

### *Pfmdr1* gene copy number

*Pfmdr1* copy number was determined using TaqMan real-time PCR (7900HT Fast Real-Time PCR system, Applied Biosystems, Courtaboeuf, France) using the single-copy gene β-tubulin (PF10_0084) as the reference. Each sample selected on the basis of a sufficient amount of DNA was genotyped in duplicate. The sequence of oligonucleotide primers and probes used, the preparation of reaction mixture, PCR conditions, and evaluation of PCR efficiency were described in detail in a previous study [21]. DNA extracted from the *P. falciparum* 3D7 reference clone, which has a single copy of each gene, was used as a calibrator, and β-tubulin housekeeping gene was used as a control in all experiments. The number of gene copy was determined using the 2^−ΔΔCt^ method [11].

### Statistical analysis

Based on similar epidemiological and ecological characteristics in the study sites of Aioun and Tintane [16], data from these two districts were pooled and compared to those from Kobeni. The frequency of a particular mutant allele was calculated as the proportion of the specific mutant samples among the total number of samples successfully analysed for this mutation. Similarly, the frequencies of double, triple, quadruple, and quintuple mutants were determined as the proportion of subjects with two, three, four, or five mutations among the total numbers of samples tested. Pairwise comparison of mutation frequencies between the northern (Aioun and Tintane) and southern (Kobeni) sites was performed using the Chi square test. The distribution of *Pfmdr1* and *Pfcrt* SNPs in relation to *Pfmdr1* copy number was compared using Fisher’s exact test. For statistical analyses, mixed alleles were considered to be mutant. The *P* value of ≤0.05 was considered as statistically significant.

## Results

The clinical characteristics of the patients and parasitological features were published elsewhere [16]. In that study, laboratory diagnosis was based on microscopy and rapid diagnostic test. *Plasmodium falciparum* mono-infection was confirmed by PCR in 299 (146 in Kobeni, 92 in Tintane, and 61 in Aioun) patients. *Plasmodium vivax* was detected by PCR in 11 additional patients (nine in Kobeni, two in Tintane, none in Aioun).

### *Pfcrt* polymorphisms

The key codon 76 was examined in the *Pfcrt* gene (Table 2). The mutant allele 76T was identified in 193 of 280 (68.9%) samples that were successfully amplified, of which 160 (57.1%) were pure mutant allele and 33 (11.8%) had mixed alleles with both K76 and 76T. The difference in the proportions of mutant and mixed alleles in Kobeni and Tintane/Aioun was not statistically significant (*P* > 0.05).

**Table 2:** Prevalence of *Pfmdr1* and *Pfcrt* point mutations in isolates from three health facilities in Hodh Elgharbi region in Mauritania

Gene	Codons	Aa	n (%)			
			Aioun	Kobeni	Tintane	Total
*Pfcrt*	K76**T**	K	12 (21)	42 (31)	33 (38)	87 (31)
		K/**T**	8 (14)	15 (11)	10 (11)	33 (12)
		**T**	36 (64)	80 (58)	44 (51)	160 (57)
*Pfmdr1*	N86**Y**	N	35 (70)	102 (77)	66 (80)	203 (77)
		**Y**	15 (30)	30 (23)	16 (20)	61 (23)
	Y184**F**	Y	14 (33)	46 (36)	20 (26)	80 (32)
		**F**	29 (67)	81 (64)	57 (74)	167 (68)
	S1034**C**	S	29 (100)	67 (96)	53 (98)	149 (97)
		**C**	0	3 (4)	1 (2)	4 (3)
	N1042**D**	N	29 (100)	76 (99)	49 (100)	154 (99)
		**D**	0	1 (1)	0	1 (1)
	D1246Y	D	37 (100)	81 (98)	70 (100)	188 (99)
		**Y**	0	2 (2)	0	2 (1)

*aa* amino acid, *n* number of isolates

### *Pfdhps* and *Pfdhfr* polymorphisms

The results of *Pfdhps* and *Pfdhfr* polymorphisms are summarized in Tables 3, 4 and 5. Of 299 available samples, *Pfdhps* PCR genotyping was successful in 264 (88.3%) samples for codons 436, 540, 581, and 613 (263 samples for codon 437). The most prevalent *Pfdhps* mutations affected the codons 436 (S436A) and 437 (A437G), which were present as pure mutants in 144 of 264 (55%) and 60 of 263 (23%) samples, respectively (Table 3). In these two codons, mixed alleles were observed in 36 of 264 (14%) and 31 of 263 (12%) samples, respectively. At the district level, samples from Kobeni exhibited the highest prevalence for S436A mutation (72.2%; *n* = 75 pure mutant allele and 16 mixed alleles) compared to Tintane and Aioun (64.5%; n = 69 pure mutant allele and 20 mixed alleles) districts, but the difference between these two areas in Hodh Elgharbi was not statistically significant (*P* > 0.05). The presence of A437G substitution is one of the hallmarks of sulfadoxine resistance. The proportion of isolates with pure or mixed A437G alleles was higher in Tintane and Aioun (61 of 137; 44%) than in Kobeni (30 of 126; 24%) (*P* < 0.05). However, K540E substitution, which together with A437G change is associated with sulfadoxine resistance, was rarely observed (2%) among the isolates collected in Hodh Elgharbi region. All isolates carried wild-type alleles in codons 581 and 613. Pure AAKAA haplotype was the most commonly observed *Pfdhps* haplotype (127 of 264, 48%), followed by SGKAA, which occurred in 71 (38 pure and 33 mixed) of 264 (26.9%) samples (Table 4).

**Table 3:** Prevalence of *Pfdhps* and *Pfdhfr* mutations in isolates collected in three health facilities in Hodh Elgharbi region, Mauritania in 2010

Gene	Codon	Aa	n (%)			
			Aioun	Kobeni	Tintane	Total
*Pfdhps*	S436**A/F**	S	17 (32)	33 (26)	32 (38)	82 (31)
		S/**A**	10 (19)	16 (13)	10 (12)	36 (14)
		**A**	26 (49)	75 (60)	43 (51)	144 (55)
		**F**	0	2 (2)	0	2 (1)
	A437**G**	A	33 (62)	96 (76)	43 (51)	172 (65)
		A/**G**	10 (19)	12 (10)	9 (11)	31 (12)
		**G**	10 (19)	18 (14)	32 (38)	60 (23)
	K540**E/A**	K	52 (98)	122 (97)	85 (100)	259 (98)
		K/**A**	0	1 (0.8)	0	1 (0.4)
		K/**E**	0	2 (2)	0	2 (1)
		**E**	1 (2)	1 (1)	0	2 (1)
*Pfdhfr*	N51**I**	N	31 (58)	73 (55)	39 (47)	143 (53)
		N/**I**	10 (19)	16 (12)	11 (13)	37 (14)
		**I**	12 (23)	44 (33)	33 (40)	89 (33)
	C59**R**	C	32 (60)	68 (51)	40 (48)	140 (52)
		C/**R**	9 (17)	18 (14)	9 (11)	36 (13)
		**R**	12 (23)	47 (35)	34 (41)	93 (35)
	S108**N/T**	S	31 (58)	68 (52)	42 (51)	141 (53)
		S/**T**	1 (2)	3 (2)	1 (1)	5 (2)
		S/**N**	7 (13)	14 (11)	6 (7)	27 (10)
		**T/N**	2 (4)	1 (1)	6 (7)	9 (3)
		**T**	2 (4)	0	1 (1)	3 (1)
		**N**	10 (19)	46 (35)	27 (33)	83 (31)
	I164**L**	I	52 (98)	131 (99)	73 (88)	256 (96)
		I/**L**	1 (2)	1 (1)	10 (12)	12 (4)

*aa* amino acid, *n* number of isolates. All isolates had the wild-type *Pfdhps* alleles in codons 581 and 613. Mutant alleles are denoted in bold characters

**Table 4:** Prevalence of wild-type and mutant *Pfdhps* haplotypes in isolates collected from three health facilities in Hodh Elgharbi region in Mauritania in 2010

Haplotypes	n (%)			
	Aioun	Kobeni	Tintane	Total
SAKAA^a^	10 (19)	18 (14)	6 (7)	34 (13)
AA/GKAA	2 (4)	2 (2)	3 (4)	7 (3)
AAKAA	22 (42)	69 (55)	36 (42)	127 (48)
AGKAA	2 (4)	4 (3)	4 (5)	10 (4)
FAKAA	0	2 (2)	0	2 (1)
S/AA/GKAA	6 (11)	9 (7)	4 (5)	19 (7)
S/AAK/EAA	0	1 (1)	0	1 (0.4)
S/AAKAA	1 (2)	6 (5)	1 (1)	8 (3)
SA/GKAA	2 (4)	1 (1)	2 (2)	5 (2)
SGEAA	1 (2)	0	0	1 (0.4)
SGK/AAA	0	1 (1)	0	1 (0.4)
SGK/EAA	0	1 (1)	0	1 (0.4)
SGKAA	4 (8)	11 (9)	23 (27)	38 (14)
Total	53 (100)	126 (100)	85 (100)	264 (100)

^a^Wild-type haplotype. The haplotypes of one isolate from Tintane (SN/AKAA, N = undetermined amino acid) and one isolate from Kobeni (SGEAN/A) were not fully characterized

**Table 5:** Prevalence of *Pfdhfr* mutant haplotypes in isolates from three health facilities in Hodh Elgharbi, Mauritania

Haplotype	n (%)			
	Aioun	Kobeni	Tintane	Total
ANCSI^a^	28 (53)	64 (48)	35 (42)	127 (47)
ANCS/**T**I	1 (2)	2 (2)	0	3 (1)
ANC/**R**SI	0	1 (1)	0	1 (0.4)
ANC/**R**S/**N**I	0	3 (2)	1 (1)	4 (1)
ANCSI/**L**	1 (2)	0	2 (2)	3 (1)
AN/**I**C/**R**S/**N**I	6 (11)	11 (8)	4 (5)	21 (8)
AN/**I**C/**R**S/**N**I/**L**	0	0	1 (1)	1 (0.4)
AN/**I**C/**R**S/**T**I	0	1 (1)	0	1 (0.4)
AN/**I**C/**R**SI	1 (2)	2 (2)	2 (2)	5 (2)
AN/**I**C/**R**SI/**L**	0	0	1 (1)	1 (0.4)
AN/**I**CS/**T**I/**L**	0	0	1 (1)	1 (0.4)
AN/**I**CSI	1 (2)	1 (1)	0	2 (1)
AN/**I**CSI/**L**	0	0	2 (2)	2 (1)
AN/**IR**NI	2 (4)	1 (1)	0	3 (1)
AN**RN**I	1 (2)	3 (2)	1 (1)	5 (2)
A**I**C/**R**S/**N**I	1 (2)	0	0	1 (0.4)
A**I**C/**RT**/**N**I	1 (2)	0	0	1 (0.4)
A**I**C**N**I	1 (2)	1 (1)	0	2 (1)
A**IRN**I	6 (11)	41 (31)	25 (30)	72 (27)
A**IRN**I/**L**	0	0	1 (1)	1 (0.4)
A**IRT**/**N**I	1 (2)	0	4 (5)	5 (2)
A**IRT**/**N**I/**L**	0	1 (1)	2 (2)	3 (1)
A**IRT**I	2 (4)	0	1 (1)	3 (1)
Total	53 (100)	133 (100)	83 (100)	269 (100)

^a^Pure wild-type *Pfdhfr* haplotype. The complete haplotype of one isolate was not determined (AIRNN, N not determined)

Of 299 samples, *Pfdhfr* PCR genotyping was successful in 269 (90.0%; codons 51 and 59) and 268 (89.6%; codons 108 and 164), respectively. The results for *Pfdhfr* polymorphisms showed that at four (51, 59, 108, 164) explored codons, pure mutant or mixed alleles were present in 126 of 269 (46.8%), 129 of 269 (48.0%), 127 of 268 (47.4%), and 12 of 268 (4.5%), respectively (Table 3). The proportions of mutant N51I, C59R and S108 N/T alleles did not differ significantly between Kobeni and Tintane-Aioun (*P* > 0.05). All isolates with mutant 164L had mixed alleles, and there were more isolates with mixed 164 alleles in Tintane and Aioun than in Kobeni (*P* < 0.05). The pure wild-type *Pfdhfr* ANCSI haplotype was identified in 64 of 133 (48.1%) and 63 of 136 (46.3%) samples in Kobeni and Tintane-Aioun (*P* > 0.05), respectively, with a global prevalence of 47.2% (127 of 269) (Table 5). A single mutant (ANC**N**I) was not observed. Pure double mutants (A**I**C**N**I or AN**RN**I) occurred rarely (seven of 269, 2.6%). The triple pure mutant haplotype, AIRNI, was detected in 41 of 133 (30.8%) and 31 of 136 (22.8%) malaria-positive samples in Kobeni and Tintane-Aioun districts (*P* > 0.05), respectively, with an overall prevalence of 27% (72 of 269).

There were three isolates with quadruple mutations (i.e., triple *Pfdhfr* mutant haplotype A**IRN**I and single *Pfdhps* mutant haplotype S**G**KAA). Quintuple mutation, i.e., S**GE**AA haplotype associated with the triple *Pfdhfr* mutant haplotype A**IRN**I, was observed in two isolates.

### *Pfmdr1* polymorphisms

A total of 264, 247, 153, 155, and 190 samples were successfully genotyped for *Pfmdr1* codons 86, 184, 1034, 1042, and 1246, respectively (Table 2). In all investigated codons, wild-type alleles predominated, except for codon 184 in which the mutant allele 184F occurred more frequently (68%; 167 of 247), compared to the wild-type allele Y184. Mixed *pfmdr1* alleles were not observed. The proportions of mutant alleles at each codon did not differ significantly (*P* > 0.05) between the northern (Tintane and Aioun) and southern (Kobeni) districts of Hodh Elgharbi region.

### *Pfmdr1* copy number

Due to insufficient DNA left after characterizing four molecular markers, the copy number of *Pfmdr1* was determined in 102 isolates (88 isolates from Kobeni and 14 from Tintane and Aioun) (Table 6). All 102 isolates were successfully genotyped. Only one isolate (one of 102, 1.0%) was characterized with three copies of *Pfmdr1*. Nine additional isolates (8.8%) had two copies of the gene. All isolates with multiple copies of *Pfmdr1* were collected in Kobeni. All other isolates (n = 92) carried a single copy of *Pfmdr1* gene. The copy number variations of *Pfmdr1* were not associated with any mutation of this gene or with *Pfcrt* SNP (*P* > 0.05).

**Table 6:** *Pfmdr1* gene copy number among *P. falciparum* isolates from three health facilities in Hodh Elgharbi, Mauritania

Number of copies	n (%)			
	Aioun	Kobeni	Tintane	Total
One	6 (100)	78 (88.6)	8 (100)	92 (90.2)
Two	0	9 (10.3)	0	9 (8.8)
Three	0	1 (1.1)	0	1 (1.0)

## Discussion

The present study was conducted during the peak season of malaria transmission in three districts in the Hodh Elgharbi region. A high prevalence of mutant K76T *Pfcrt* allele was observed in the present study. This finding is in agreement with the previous clinical and molecular studies, confirming that *Pfcrt* is useful for the detection and surveillance of chloroquine-resistant *P. falciparum* in Hodh Elgharbi region [13].

The present study also revealed the high prevalence of Y184F and, to a lower extent, N86Y mutations in *Pfmdr1* gene. It has been suggested that *Pfmdr1* mutations may play a role in modulating the levels of resistance to several drugs [23]. Amodiaquine treatment failure has been associated with the selection of *P. falciparum* isolates carrying the mutant *Pfcrt* 76T allele and *Pfmdr1* haplotype 86Y, Y184, and 1246Y [23–26]. In the present study, the majority of isolates (68.9% with either pure mutant or mixed alleles) had the mutant *Pfcrt* 76T allele, but the *Pfmdr1* mutant haplotype associated with amodiaquine resistance was rare (for 1246Y mutant, two of 190 isolates, 1.0%). These molecular findings are in agreement with the high clinical efficacy of artesunate-amodiaquine in Kobeni (98.2% efficacy on day 28 after PCR correction) and the fact that amodiaquine monotherapy had not been used before adoption of ACT in the country [2]. An opposite trend for the selection of *P. falciparum* isolates with *Pfmdr1* haplotype N86, 184F, and D1246 has also been reported after artemether-lumefantrine treatment [8–10, 26]. Since artesunate-amodiaquine and artemether-lumefantrine are widely employed to treat *P. falciparum* in Mauritania, further surveillance of ACT resistance can be pursued using the combination of *Pfcrt*, *Pfmdr1* and *kelch 13* as surrogate markers for resistance to these drugs, in parallel with regular clinical evaluation of ACT efficacy. The limitations of the study on *Pfmdr1* include the lack of complete molecular data for all isolates due to sub-optimal PCR conditions for the fragment spanning codons 1034, 1042, and 1246, the limited quantity of DNA available, and possible degradation of DNA due to poor storage conditions. The present study was conducted before the discovery of *kelch 13*, and insufficient blood sample did not allow further study on this novel marker.

*Pfmdr1* gene copy number is pertinent for monitoring possible emergence of resistance to amino alcohols (lumefantrine and mefloquine), which is also influenced by *Pfcrt* and *Pfmdr1* alleles [8–10]. At present, the proportion of isolates with increased gene copy number is limited in Mauritania, but further monitoring is required, in particular in Kobeni, where isolates with multiple copies of *Pfmdr1* gene were found. In addition, an evaluation of the efficacy of artemether-lumefantrine in Kobeni is necessary to establish baseline clinical data.

*Pfdhps* A437G substitution is one of the components of the ‘quintuple *Pfdhps*-*Pfdhfr* mutations’ associated with SP resistance [27]. The analysis of molecular markers in the present study revealed the presence of 437G in 23% (35% including mixed alleles) of the isolates: 19% (38% including mixed alleles) in Aioun and 14% (24% including mixed alleles) in Kobeni. In an earlier study conducted in 1998, there were 22 and 16% of mutant 437G in Aioun and Kobeni, respectively [14]. There was only a slight increase in the proportion of mutant *Pfdhps* 437G allele in Kobeni after 12 years. By contrast, the proportion of mutant 437G allele almost doubled during the same period in Aioun. However, the other *Pfdhps* component of the ‘quintuple mutant’, K540E, was observed in only four of 264 (1.5%) isolates in the present study and none in the 1998 study [14].

Triple *Pfdhfr* mutant (AIRNI) was present in a total of 106 of 269 (39%) isolates, either as pure alleles (n = 72, 27%) or mixed alleles (n = 34, 12.6%). In an earlier study, Eberl et al. reported 16.9% (ten of 59 isolates) and 12.6% (13 of 103 isolates) of triple *Pfdhfr* mutants in Aioun and Kobeni, respectively [14]. The proportions of triple *Pfdhfr* mutants increased two- to three-fold in Aioun and Kobeni between 1998 and 2010. This observation is consistent with the frequent use of SP for the treatment of uncomplicated malaria until 2006 and the continuous use of this drug for intermittent preventive treatment in pregnant women since 2006 [28]. The progression of *Pfdhps* mutants overtaking wild-type isolates between 1998 and 2010 seems to be slow in Kobeni compared to Tintane and Aioun where mutations in both *Pfdhps* and *Pfdhfr* occurred at a similar rate between 1998 and 2010. Quintuple mutant occurred rarely in the present study. Moreover, *Pfdhfr* I164L substitution, which confers a high level of pyrimethamine resistance, was found as mixed alleles in few isolates. The results of the molecular assays suggest that SP is still useful in targeted population (i.e., in pregnant women and possibly in infants) for intermittent preventive treatment. Other studies have suggested that intermittent preventive treatment with SP remains effective for fetal and maternal protection even in areas of high resistance to SP [29].

Current knowledge on malaria epidemiology is still inadequate to develop a concerted plan to control cross-border malaria in the study area. In addition to clinical and molecular studies on drug-resistant malaria and entomological surveys, sociological studies are needed to understand the patterns of human population movement of the nomads, refugees and local travellers along and across the Malian–Mauritanian border. These difficulties are compounded by the presence of *P. vivax*, which is notoriously difficult to eliminate due to hypnozoites, requiring primaquine for radical cure. One recent study failed to detect *P. vivax* along the Malian–Mauritanian border (Selibaby, Ould Yenge, Aioun, Kobeni, Timbedra, Nema) [30]. However, the results of other studies, including the present study, indicate the presence of *P. vivax* in this hotspot [15, 16, 31]. A coordinated regional malaria control programme will be required in the effort to control cross-border malaria that involves both *P. falciparum* and *P. vivax* [32].

## Conclusions

Despite the introduction and use of ACT in Mauritania since 2006, the prevalence of genetic polymorphisms associated with drug resistance reached levels of concern. Although the prevalence of quintuple *Pfdhfr*-*Pfdhps* mutants was low in the present study, the high percentage of triple *Pfdhfr* mutants associated with the key *Pfdhps* A437G mutation, the high prevalence of mutant *Pfcrt* allele, and, to a lesser extent, the observed mutations in *Pfmdr1* gene may jeopardize the future of seasonal malaria chemoprevention based on amodiaquine-SP and the use of SP for intermittent preventive treatment in pregnancy. Further molecular studies using these molecular markers, in parallel with clinical evaluation of currently deployed anti-malarial drugs, are warranted to monitor and anticipate the possible spread of drug-resistant *P. falciparum* in the malaria hotspot along the Malian–Mauritanian border. A new marker, *kelch 13*, should be added in future molecular surveillance activities for a more complete data collection.

## Abbreviations

ACT: artemisinin-based combination therapy
DHFR: dihydrofolate reductase
DHPS: dihydropteroate synthase
EDTA: ethylenediaminetetraacetic acid
*Pfcrt*: *Plasmodium falciparum* chloroquine resistance transporter
*Pfdhfr*: *Plasmodium falciparum* dihydrofolate reductase gene
*Pfdhps*: *Plasmodium falciparum* dihydropteroate synthase gene
*Pfmdr1*: *Plasmodium falciparum* multidrug resistance gene 1
RFLP: restriction fragment length polymorphism
SNP: single nucleotide polymorphism
SP: sulfadoxine-pyrimethamine
